# Determinants of Anemia among School-Aged Children in Mexico, the United States and Colombia

**DOI:** 10.3390/nu8070387

**Published:** 2016-06-23

**Authors:** Sana Syed, O. Yaw Addo, Vanessa De la Cruz-Góngora, Fayrouz A. Sakr Ashour, Thomas R. Ziegler, Parminder S. Suchdev

**Affiliations:** 1 Department of Pediatrics, Emory University School of Medicine, Atlanta, GA 30322, USA; syedsana@gmail.com; 2 Hubert Department of Global Health, Emory University Rollins School of Public Health, Atlanta, GA 30322, USA; yaw.addo@emory.edu; 3 Division of Nutrition and Health, Instituto Nacional de Salud Pública, Cuernavaca, Morelos 62100, Mexico; vcruz@insp.mx; 4 Department of Nutrition & Food Science, College of Agriculture and Natural Resources, University of Maryland, College Park, MD 20742, USA; Fayrouz.Ashour@gmail.com; 5 Department of Medicine, Emory University School of Medicine, Atlanta, GA 30322, USA; tzieg01@emory.edu

**Keywords:** anemia, iron deficiency, school aged children, Mexico, United States, Colombia

## Abstract

Anemia affects approximately 25% of school-aged children (SAC—aged 5.00–14.99 years) globally. We determined in three countries the prevalence and determinants of anemia in SAC. Data on sociodemographics, inflammation and nutrition status were obtained from the 2006 Mexican National Nutrition Survey, the 2003-6 US National Health and Nutrition Examination Surveys, and the 2010 Encuesta Nacional de Nutrición Situación Colombia. In the US, vitamin A and iron deficiency (ID) were available only for girls aged 12.00–14.99 years to which our analysis was limited. Associations were evaluated by country using multivariable logistic regression adjusting for confounders and complex survey design. The prevalence of anemia and ID were: Mexico 12% (ID 18%), *n* = 3660; US 4% (ID 10%), *n* = 733; and Colombia 4% (ID 9%), *n* = 8573. The percentage of anemia associated with ID was 22.4% in Mexico, 38.9% in the US and 16.7% in Colombia. In Mexico, anemia was associated with ID (adjusted OR: 1.5, *p* = 0.02) and overweight (aOR 0.4, *p* = 0.007). In the US, anemia was associated with black race/ethnicity (aOR: 14.1, *p* < 0.0001) and ID (aOR: 8.0, *p* < 0.0001). In Colombia, anemia was associated with black race/ethnicity (aOR: 1.6, *p* = 0.005), lowest socio-economic status quintile (aOR: 1.8, *p* = 0.0005), ID (aOR: 2.7, *p* < 0.0001), and being stunted (aOR: 1.6, *p* = 0.02). While anemia was uniformly associated with iron deficiency in Mexico, Columbia, and the United States, other measured factors showed inconsistent associations with anemia. Additional data on anemia determinants in SAC are needed to guide interventions.

## 1. Introduction

Anemia is characterized by low hemoglobin (Hb) concentration, red-cell count, or packed-cell volume, with subsequent impairment in meeting the oxygen delivery to demands of tissues. Hb concentration and thus anemia is affected by personal/individual characteristics such as age, sex, and pregnancy status, as well as environmental factors such as smoking and altitude [1]. Anemia is currently estimated to impact a quarter of the world’s population [2]. Anemia has been shown to contribute to mortality; a recent meta-analysis of nearly 12,000 children from six African countries aged 28 days to 12 years indicates that for each 1 g/dL increase in Hb, the risk of death falls by 24% [3]. The 2013 Global Burden of Disease study [4] assessed the leading causes of global years lived with disability (YLDs) from 1990 to 2013 and estimated that iron deficiency anemia (IDA) was the leading cause of YLDs among children and adolescents, affecting 619 (95% uncertainty interval, 618–621) million in 2013. The 50 countries with the largest child and adolescent population contributed to 86% of global iron deficiency anemia cases in this population. India contributed the largest number of cases (147.9 million), followed by China (75.8 million) and Nigeria (24.7 million) [4]. Other main causes of anemia are: infectious diseases such as malaria, HIV, hookworm infections and schistosomiasis; deficiencies of other key micronutrients, including vitamin A, folate, and vitamin B12; inherited blood disorders, such as thalassemia; and anemia of chronic disease [5].

The prevalence of anemia varies significantly by sex and age with an estimated prevalence in the most vulnerable populations ranging from 47% in pre-school children (PSC), 42% in pregnant women, 30% in non-pregnant women, and 25% in school aged children (SAC) [2]. Across age groups, long-term anemia is associated with loss of productivity from impaired work capacity, cognitive impairment, and increased susceptibility to infection [6]. Iron deficiency (ID) in infancy has long-lasting effects on brain development, including cognitive, motor and social-emotional function [7]. Given that ID is thought to be one of the main causes of anemia worldwide [8], most anemia interventions globally involve iron supplementation [9].

The role of inflammation in the context of correctly interpreting micronutrient status is an area of avid interest [10] as several nutrient biomarkers including measures of iron deficiency (e.g., serum ferritin and soluble transferrin receptor) are affected by inflammation and can lead to underestimation of the true disease burden. Given the higher prevalence of anemia in PSC and pregnant women, most research has been focused on these two populations [2]. Current estimates derived from data from 36 countries represent only 33% of the global population of SAC [11] making this age group a research priority. Furthermore, there is a need to refine the relative contribution of nutritional and other risk factors for anemia across different geographic settings to better guide national anemia reduction programs.

In this study, we determined the prevalence of anemia and ID among SAC aged 5.00–14.99 in Mexico and Colombia, and among girls aged 12.00–14.99 in the USA. We also investigated the degree to which select known anthropometric, biochemical, demographic and socio-economic determinants were associated with anemia in each country.

## 2. Materials and Methods

### 2.1. Study Population and Sample

In 2011, the Centers for Disease Control and Prevention (CDC), Global Alliance for Improved Nutrition and Eunice Kennedy Shriver National Institute of Child Health and Human Development formed a collaborative research group called Biomarkers Reflecting Inflammation and Nutrition Determinants of Anemia (BRINDA). Details of the BRINDA project objectives and methodology have been previously published [12]. In brief, data from nationally and regionally representative surveys conducted after 2004 that included PSC (aged 6.00–59.99 month), SAC (aged 5.00–14.99 years) and women of reproductive age (WRA, aged 15.00–49.99 years) and reported a minimum of the following biomarkers: (1) Hb; (2) a measure of inflammation (C-reactive protein, (CRP) and/or α-1-acid glycoprotein, (AGP)); and (3) a measure of iron status (ferritin and/or soluble transferrin receptor, (sTFR)). A total of 23 datasets were identified. Permission was received for 16 datasets from which SAC information was available from national cross-sectional surveys from the following three countries—Mexico, the US and Colombia. For each country, the survey years included for analysis were based on availability of data. When possible, the most recent surveys available were also utilized.

In Mexico, analyses were based on data from the Mexican National Health and Nutrition Survey 2006 (ENSANUT 2006), a nationally-representative, multi-stage, stratified cluster sampling survey conducted between October 2005 and May 2006. A detailed description of the design and survey methodology of the ENSANUT 2006 has been published elsewhere [13]. Data for the US was obtained from two continuous cycles of The National Health and Nutrition Examination Surveys (NHANES), 2003 to 2006. This is a complex, multistage probability sample of the US civilian, non-institutionalized population. A detailed description of the sampling procedures and survey methodology of NHANES 2003–2006 has been published previously [14,15]. In NHANES 2003–2004 and 2005–2006, ferritin (*n* = 1056) and sTFR (*n* = 1038) information was only available for children aged 5 years in both sexes, and for adolescent girls aged 12.00–14.99 years. Thus, we limited our analysis in US to adolescent girls aged 12.00–14.99 (*n* = 733). In Colombia, analyses were based on data from the 2010 Encuesta Nacional de la Situaci ón Nutricional en Colombia (ENSIN). This was a nationally-representative nutritional survey in both rural and urban settings, from 2008–2010. A detailed description of the sampling procedures and survey methodology of the ENSIN 2010 has been previously published [16].

**Figure 1:**
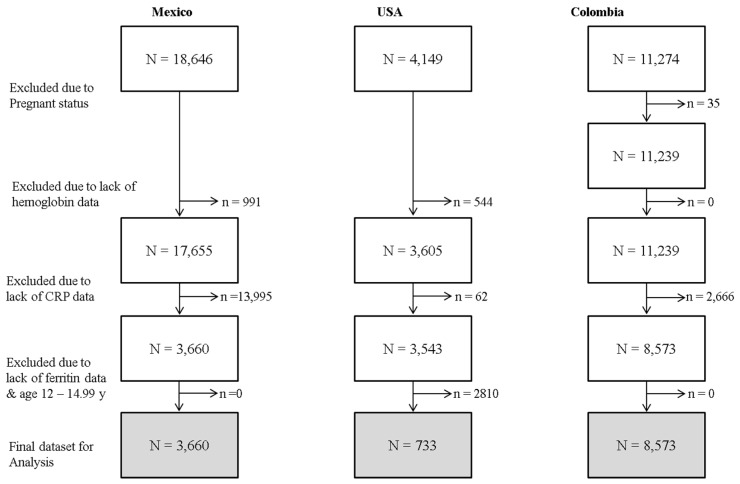
Sample selection for analysis of anemia in school aged children aged 5.00–14.99 years in Mexico and Colombia, and adolescent girls aged 12.00–14.99 years in the USA.

The overall sample size of SAC available in each country was 18,646 in Mexico, 4149 in the US and 11,274 in Colombia (Figure 1). We restricted our analysis to subjects with the following biomarker data: Hb (*n* removed due to missing data: Mexico = 991, USA = 544, Colombia = 0), ferritin (*n* removed due to missing data: Mexico = 0, USA = 2810, Colombia = 0) and CRP (n removed due to missing data: Mexico = 13,995, USA = 62, Colombia = 2666). Thus, data were analyzed for 3660 Mexican and 8573 Colombian children aged 5.00–14.99 years; US data was analyzed for 733 SAC adolescent girls aged 12.00–14.99 years (Figure 1).

From each country survey, we selected children who met the following inclusion criteria: (1) age of the participant ≥5 years and ≤14.99 years; (2) availability of Hb level as a measure of anemia; (3) availability of CRP as a measure of inflammation; and (4) availability of serum ferritin as a measure of iron status. From each country survey, we excluded pregnant participants. We further limited our US sample to adolescent girls aged 12–14.99 years.

### 2.2. Assessment of Nutrition and Health Status

The primary outcome for these analyses was anemia, which was measured using hemoglobin (Hb). The following thresholds for anemia were used as per World Health Organization (WHO) guidelines [2]; Hb < 11.5 g/mL for children aged 5.00–11.99 years and Hb < 12.0 g/mL for children aged 12.00–14.99 years. Anemia was adjusted based on the altitude of the cluster in which a participant resided. This adjustment was based on subtracting published altitude adjustment for specific Hb concentrations [17] from the Hb values observed for participants from Mexico and Colombia. Altitude information was not available in the US. Anemia in Colombia was also adjusted for smoking [18,19] based on subtracting published smoking adjustment Hb concentrations [17]. No smoking information for SAC was available in the US and Mexico.

The following data were collected: demographics (age, sex and race/ethnicity), socio-economic status (SES), ferritin as a measure of iron status, and CRP as a measure of inflammation. Data on sTFR as a measure of iron status was available only in Mexico and the US. Retinol as a measure of vitamin A status was available only in the US. Principal component analysis (PCA) was used to classify respondents’ SES [20] into a 5-quintile asset index based on household durable goods in Mexico and Colombia. In the US, continuous poverty income ratio (PIR) was categorized into five quintiles to create an asset variable. The PIR, for the US, is a ratio of a family income to the poverty threshold for that size family based on the US National Census bureau [21]. For all countries, low socioeconomic status was defined as the poorest quintile; therefore SES was categorized into two groups: poorest quintile versus all other quintiles. Race information was available in the US and Colombia and was dichotomized as black versus non-black racial groups. Original categories in each country were as follows: Mexico—no race/ethnicity information available; US—Mexican American, other Hispanic (this would include those who identified as Hispanic Black), Non-Hispanic White, Non-Hispanic Black and other Race, including Multi-racial; and Colombia—Indigenous, Gypsy or Roma, Raizal Archipelago, Palenquero San Bacillus, Black/Mulatto/Afro-Colombian/Afro, and none of the above. Age was categorized into two groups using a cut-off of 12.0 years—this was a biologically determined cut off for mean age of menarche in all three countries using prior literature [22,23,24,25].

The following thresholds were used to define abnormal values for these biochemical indicators: (1) ferritin < 15 mg/L [26]; (2) sTFR > 8.3 mg/L [27,28]; (3) retinol < 0.7 mmol/L [29]; and (4) CRP > 5 mg/L [30]. Ferritin was used to measure ID because it has the highest sensitivity and specificity to detect iron deficiency in those without inflammation in comparison to bone marrow iron [31]. As ferritin and retinol are acute phase proteins, these biomarkers are affected by the presence of inflammation with ferritin increasing and retinol decreasing, and thus underestimating and over estimating prevalence of deficiency respectively. Therefore, ID and vitamin A deficiency were calculated excluding individuals with elevated CRP. Exclusion was used to account for inflammation rather than other approaches because of the low prevalence of inflammation in our samples as measured by elevated CRP and the lack of a second measure of chronic inflammation (e.g., AGP) [32]. IDA was defined as the presence of anemia along with low ferritin among those with CRP ≤ 5 mg/L [28,33]. Further details of the laboratory methods in each country are outlined in Table S1. In regression models CRP was treated as a covariate and as such, those with inflammation were not excluded. We also calculated the % of anemia associated with ID as follows: % IDA divided by % anemia.

Measures of anthropometrics included measurements of weight and height using standardized techniques by trained health workers for all three surveys. Details of the laboratory analyses from each country have previously been described [13,14,15,16] and are outlined in the online Supplementary Materials (Table S1). Of note, different methods of Hb measurement were used in each country. In Mexico, the concentration of Hb in capillary blood was measured by finger prick using a portable photometer. The determination of Hb was made using a HemoCue (Angelholm, Sweden) [34,35,36]. Similarly, in Colombia, Hemoglobin was measured by the HemoCue method (HemoCue AB) [37]. In the NHANES, hemoglobin was measured as part of the complete blood count using the Beckman Coulter™ HMX Hematology Analyzer (Brea, CA, USA) [14], which is a more precise method.

### 2.3. Data Management and Statistical Methods

Statistical analyses were done using SAS 9.3 (SAS Institute Inc., Cary, NC, USA) and R 3.1.1 (R Foundation for Statistical Computing, Vienna, Austria). Statistical significance was set at a 2-sided alpha of 0.05. All analyses were adjusted for complex survey design effects using cluster, strata and weight statements. Sampling weights defined/derived in the original country surveys were used in all analyses. For each country, biomarker specific weights were used and for all Hb analysis (including logistic regression for anemia); the Hb specific weight was used as the sampling weight variable. Participant descriptive statistics were presented as means (standard error (SE)) and as percentage (95% confidence interval (CI)) for continuous and categorical outcomes. Crude prevalence and 95% CIs for anemia in each of the study population, was examined using SAS PROC SURVEYFREQ to account for complex survey design effects. We used the WHO Child Growth Standards (WHO Anthro, Geneva, Switzerland) [38] to calculate *z*-scores, and categorized stunting as a height-for-age *z*-score < −2 SD (standard deviation), wasting as a weight-for-height *z*-score < −2 SD, overweight as a BMI-for-age *z*-score > 2 SD and obesity as a BMI-for-age *z*-score > 3 SD.

### 2.4. Multivariable Modeling Approach

**Figure 2:**
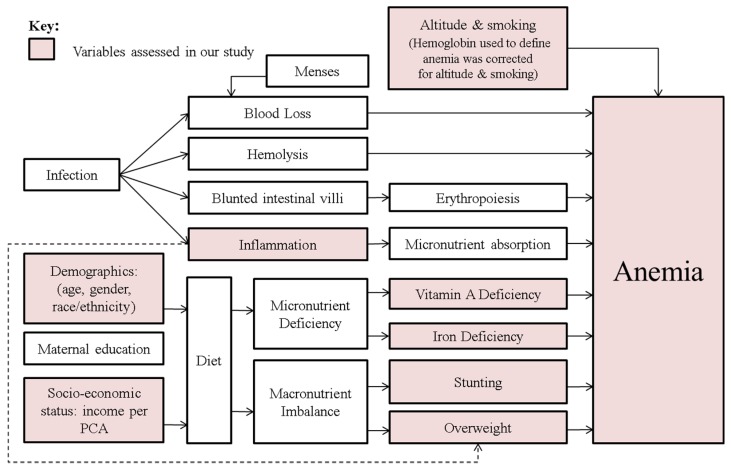
Important causal pathways for anemia among school aged children aged 5.00–14.99 years.

Determinants of anemia were evaluated using multivariable logistic regression models with adjustment for potential confounders. Anemia, defined by altitude and smoking-corrected Hb, was the dependent variable. The following covariates were considered for the multivariable model a priori based on known risk factors for anemia: categorized age, asset score, elevated CRP, low ferritin, overweight, stunting, race/ethnicity (in USA and Colombia) and vitamin A deficiency (in USA). Our multivariable modeling approach was informed by a hypothesized conceptual causal diagram (Figure 2) for anemia determinants. We computed our regression model grounded on specified/forced variables using our conceptual framework, instead of backwards elimination or other modeling approaches.

Categorized age was used in Mexico and Colombia, while in the US, age in years was used as a continuous variable and sex was excluded in the US as all were adolescent girls aged 12.00–14.99 years. Inflammation as measured by CRP was initially used in the logistic model as a categorical predictor but was not found to be significantly associated with anemia. In the final multivariable analysis, CRP was included as a continuous predictor and was also not found to be significantly associated with anemia in any country.

### 2.5. Ethics Statement

The study was reviewed by the Emory University institutional review board and deemed to be non-human subjects’ research. Each of the individual country surveys had their own ethical and human subject approvals.

## 3. Results

### 3.1. Demographic and Health Characteristics

We examined basic demographic characteristics (age, height, weight and sex) among SAC survey participants of both sexes in our original datasets as well as the final datasets used for analysis for Mexico and Colombia to evaluate for selection bias. In Mexico, age, height, weight and sex were similar in both the original and final datasets. In Colombia, age, height and weight were similar in the original and final datasets, but the percentage of girls was higher in the final dataset that was used for analysis (Table S2). Since our US analysis was considerably age and sex restricted (girls aged 12.00–14.99 years), we did not compare demographics between the original and final sample.

Mexican data (Table 1) consisted of a higher proportion of girls (59.5%) than boys. Prevalence of stunting was 10.2%, wasting was 1.3%, overweight was 13.3% and obesity was 3.9%. There was no race/ethnicity data available in the Mexican survey. The mean (SE) of Hb was 13.8 (0.6). The prevalence of anemia using Hb adjusted for altitude was 11.6%, ID was 18.1%, and IDA was 2.6%. The US data had all girls (100%). Anthropometric measures were as follows: stunting 1.6%, wasting 2.4%, overweight 19.9% and obesity 3.6%. The proportion of SAC adolescent girls with black race/ethnicity was 15.6%. The mean (SE) of Hb was 13.7 (0.7). The prevalence of anemia was 3.6%, ID was 9.7% and IDA was 1.4%. The Colombian data consisted of 57.4% girls, with anthropometric measures as follows: stunting (9.8%), wasting (2.0%), overweight (4.3%) and obese (0.5%). The mean (SE) of Hb was 14.5 (0.3). The prevalence of anemia using Hb adjusted for altitude and smoking was 4.2%, of IDA was 0.7% and of ID was 9.2%. The percentage of anemia associated with ID in each country was as follows: 22.4% in Mexico, 38.9% in the US and 16.7% in Colombia.

### 3.2. Characteristics Associated with Anemia

In multivariable analysis, after adjusting for potential confounding factors (listed in Table 2, age, sex, race, asset index, CRP and nutrition biomarkers), SAC characteristics associated with anemia in each country were: in Mexico, low ferritin (OR: 1.5; 95% CI: 1.1, 2.0) and overweight (OR 0.4; 95% CI: 0.2, 0.8); and in Colombia, black race/ethnicity (OR: 1.6; 95% CI: 1.2, 2.3), poorest SES quintile (OR: 1.8; 95% CI: 1.3, 2.5), low ferritin (OR: 2.7; 95% CI: 1.96, 3.8) and stunting (OR: 1.6; 95% CI: 1.1, 2.3). Among girls aged 12 to 14.99 years in the US (Table 3), characteristics associated with anemia included black race/ethnicity (OR: 14.1; 95% CI: 4.7, 42.1) and low ferritin (OR: 8.0; 95% CI: 3.0, 21.3).

## 4. Discussion

While there is recognition across government and stake-holder platforms of the need for good health and nutrition of SAC, this has not translated into major policy action [11]. There is a paucity of data on the nutritional status of SAC with most research and programmatic guidance focusing on children <5 years [11]. This study used data from three national nutrition surveys to assess anemia and its determinants in a sample of nearly 16,000 SAC.

We found that the prevalence of anemia in SAC in Mexico and Colombia was 11.6% and 4.2%, respectively; anemia among adolescent girls in the US was 3.6%. On a population level this prevalence of anemia translates into millions of SAC, likely accessible for public health interventions. Not only is this one of the first studies to assess determinants of anemia in SAC across several nationally-representative surveys, it is also one of the first studies to show country comparisons using standardized methods to account for inflammation. Our anemia prevalence estimates concur with previously published data from the ENSAUT 2006 (Mexico), NHANES 2004-6 (the US) and the ENSIN 2010 (Colombia) [39,40,41]. As expected, our anemia prevalence estimates were lower than from low-income and high-infection burden countries such as Haiti (anemia prevalence of 70.6% in children aged 3–13 years) [42], eastern Ethiopia (anemia prevalence of 27.1% in children aged 5–14 years) [43], and southwest Ethiopia (anemia prevalence of 37.6% in children aged 6–14 years) [44]. However, all of these estimates were derived from sub-national surveys.

In our study, we found a low prevalence of chronic malnutrition (as indicated by stunting) but a high prevalence of overweight/obesity in all three countries. Given that Mexico and Colombia are currently both considered to have upper-middle-income economies as defined by the World Bank [45], we propose that these are signs of a nutrition transition [46]. For example, in Mexico, there was the double burden of stunting (10.2%), reflective of malnutrition in early life, coexisting with an almost equal prevalence of overweight (13.3%). This may in part, be due to excessive intake of calories/food types, lifestyle choices and genetic predisposition. SAC in Colombia had a higher burden of stunting (9.8%) relative to those who were overweight (4.3%). Given the rapidity with which traditional diets and lifestyles are changing in many resource-poor countries, it is not surprising that food insecurity and under-nutrition persist in the same countries where chronic diseases such as obesity are emerging as a major epidemic [47]. This double burden of diseases [48,49] in low- and middle-income countries is well recognized such as in Mexico [50] and Latin America [46,51]. This double burden of diseases has also been shown to exist among certain subpopulations in high income countries such as among Hispanic children in the US [52].

We identified several potentially modifiable factors associated with anemia including ID as measured by low ferritin in all countries, and overweight in Mexico and stunting in Colombia. ID is a well-known modifiable determinant of anemia in children, both in the pre-school age group [53,54] and in SAC [55,56]. In these studies, anemia in girls was thought to be secondary to blood loss (and subsequent iron deficiency). This blood loss was due to infections such as malaria and schistosomiasis in younger girls (12–13 years), and menstruation in older girls (14–18 years). Poor dietary intake of iron and the low bioavailability of non-heme iron in resource-poor countries is also considered as a reason for low body iron and resultant anemia [57,58]. The US SAC adolescent female data showed 2.4% wasting, this was lower than previously published report of 10.7% among Japanese female seventh-graders, another high income country [59].

Overweight status was found to be inversely associated with anemia in Mexican SAC. In our study, among US adolescent girls aged 12.00–14.99 years, being Black was a non-modifiable characteristic associated with anemia. In Colombia being Black and being in the poorest SES quintile were non-modifiable characteristics associated with anemia. Other potentially important determinants of anemia in that were not measured in these countries include maternal education (as a contributor to SES), dietary intake of iron, and inherited blood disorders [60] and other micronutrient deficiencies including vitamin A, B12 and folate [42].

There were several strengths of our study, including: (1) updated anemia and iron deficiency estimates for Mexico and Colombia; (2) application of standard criteria across surveys from Mexico and Colombia to adjust Hb for altitude; and (3) use of similar age cut-offs in Mexico and Colombia to define SAC. Limitations of the study are primarily related to availability of data. First, we were limited by the large number of children without CRP data in Mexico (*n* = 13,995) (Table S2). Per country survey design, heights and weights were available on a representative subset of randomly selected children. This could have led to possible selection bias; however, a similar random selection method was applied to select representative children on whom biomarkers were measured which we believe prevents this bias; Second, the cross-sectional designs of all three country surveys prevented us from establishing a temporal sequence with regards to potential determinants and the development of anemia. In our multivariable models, the prevalence odds ratio that we measured is known to overstate the prevalence ratio [61] but this is a standard analysis method in large national surveys such as these. Third, different Hb methodologies were used in each country. In the US, the Hb standard deviation (SD) was the smallest, likely secondary to the Coulter™ HMX Hematology Analyzer, which is a more precise method [62]. We think that the higher Hb SDs reported from SAC in Mexico and Colombia were likely due to the different measurement methodology used, the HemoCue, which utilizes capillary, not venous blood samples. However, studies comparing Hb values obtained by the HemoCue system with those from the Coulter have found the two methods to be highly significantly correlated; given the convenience and ease of use of the Hemocue, it has been recommended by the WHO for use in field surveys where accurate and rapid anemia estimates are required [35,63]. Fourth, as outlined in the online Supplementary Materials, each country also had differences in non-Hb laboratory methodology, which also limited cross-country comparisons. Fifth, incomplete inflammation and micronutrient biomarkers were available across the three surveys. For example, sTFR as a measure of iron status was available only in Mexico and the US; retinol as a measure of vitamin A status was available only in the US. In addition, CRP was the only available inflammation biomarker. A measure of chronic such as a-1-acid glycoprotein (AGP) was not measured. CRP levels typically increase within 10 h of the onset of acute inflammation and quickly normalize within one week [64], whereas AGP levels begin to increase 24 h after the onset of inflammation but remain elevated well into convalescence [65]. Even though SES was based on PCA in each country, it was however more subjective in Mexico and Colombia where the PCA was derived from household possessions and durable goods. In comparison, the US used a more objective measure such as the poverty income ratio. Lastly, we were unable to account for other known determinants of anemia in our multivariable analysis such as information about disease chronicity, menstruation in females, inherited hemoglobinopathies, and other hematologic/oncologic disorders.

## 5. Conclusions

We conclude that ID (as defined by having low ferritin) was uniformly associated with anemia and is a modifiable factor to target anemia interventions. At risk SAC belonging to the lowest SES quintile and Black race/ethnicity may particularly be targeted. Although anemia may not affect a large percentage of SAC, it has the potential to continue into adolescence, impact school performance, increasing absenteeism and possibly affecting workforce economic potential [1]. In our study, in each of the three countries, Mexico, the US, and Colombia, low ferritin (ID) was strongly associated with anemia. School-based programs to screen and treat children for iron deficiency and anemia need to be further evaluated. Future research directions/recommendations include: (1) study other racial and ethnic groups within middle and high income countries that may face a double burden of malnutrition; (2) conduct longitudinal follow up studies that contain both CRP and AGP; (3) include measurements of both CRP and AGP in survey data; (4) include specific measures of parasitic infection; (5) collect data on blood loss/menstruation in adolescent girls; and (6) use standardized laboratory methods while conducting national surveys to minimize heterogeneity and allow better comparison across countries.

## Figures and Tables

**Table 1:** Anthropometric and biochemical characteristics of school aged children 5–14.99 years in Mexico and Colombia & adolescent girls aged 12.00–14.99 years in the USA.

Characteristics	Mexico/*n* = 3660		USA/*n* = 733		Colombia/*n* = 8573	
	*n* *	% (SE of %) or Mean (SE)	*n* *	% (SE of %) or Mean (SE)	*n* *	% (SE of %) or Mean (SE)
*Demographics*						
Age in years	3660	9.1 (0.1)	733	13.6 (0.04)	8573	9.9 (0.04)
Age						
<12.0 years	3083/3660	78.7% (1.2)	-	-	6223/8573	72.5% (0.6)
12.0–14.99 years	577/3660	21.3% (1.2)	733/733	100% (0.0)	2350/8573	27.5% (0.6)
Sex (Females) %	2115/3660	59.5% (1.6)	733	100% (0.0)	4944/8573	57.4% (0.7)
Race/Ethnicity						
Black	N/A	N/A	244/733	15.6% (2.1)	958/8573	11.6% (0.5)
Non-Black	N/A	N/A	489/733	84.4% (2.0)	7615/8573	88.4% (0.5)
Asset						
Poorest	1191/3656	31.1% (1.8)	160/717	14.3% (1.8)	3299/8573	25.9% (0.8)
All Other	2465/3656	68.9% (1.8)	557/717	85.7% (1.8)	5274/8573	74.0% (0.8)
*Nutrition/Growth*						
Stunting % (HAZ < −2)	396/3658	10.2% (0.9)	8/725	1.6% (0.7)	963/8390	9.8% (0.5)
Wasting % (BAZ < −2)	54/3658	1.3% (0.3)	8/725	2.4% (1.2)	160/8390	2.0% (0.2)
Overweight % (BAZ > 2)	450/3658	13.3% (1.3)	169/725	19.9% (2.9)	339/8390	4.3% (0.3)
Obese % (BAZ > 3)	106/3658	3.9% (0.8)	36/725	3.6% (0.8)	40/8390	0.5% (0.1)
*Biochemical markers*						
Hemoglobin (g/dL)	3660	13.8 (0.6)	733	13.7 (0.7)	8573	14.5 (0.3)
^1^ Hemoglobin (g/dL) adjusted for altitude and/or ^2^ smoking	3660	13.3 (0.5)	N/A	N/A	8573	14.1 (0.3)
C-Reactive Protein (ng/mL)	3660	2.2 (0.2)	733	1.7 (0.4)	8573	2.5 (0.1)
^2^ Iron Deficiency %	626/3302	18.1% (1.2)	^‡^ 87/680	9.7% (1.3)	736/7450	9.2% (0.4)
^3^ Low Ferritin %	663/3650	17.3% (1.2)	^‡^ 92/733	9.3% (1.3)	831/8573	9.2% (0.4)
^4^ High sTFR %	664/3645	19.6% (1.3)	^‡^ 49/727	5.0% (1.0)	N/A	N/A
^5^ Vitamin A Deficiency %	N/A	N/A	^‡^ 2/720	0.1% (0.1)	N/A	N/A
^6^ Elevated C-Reactive Protein (ng/mL) %	353/3660	9.4% (0.8)	53/733	6.9% (1.2)	1123/8573	13.2% (0.6)
^7^ Anemia %	454/3660	11.6% (0.9)	49/733	3.6% (0.8)	452/8573	4.2% (0.3)
^8^ Iron Deficiency Anemia using adjusted Hb %	103/3302	2.6% (0.4)	^‡^ 16/680	1.4% (0.5)	77/7450	0.7% (0.1)
^9^ Anemia associated with Iron Deficiency %	-	22.4%	-	38.9%	-	16.7%

* Reported n is of actual sampled population. Reported % are weighted per the survey design, Abbreviations: Height for Age *z* score—HAZ; Body Mass Index for Age *z* score—BAZ; soluble transferrin receptor—sTfR. ^1^ Hemoglobin values adjusted for altitude (for Mexico & Colombia) and for smoking (Colombia) [17]; ^2^ Iron Deficiency % assessed using serum ferritin (SF) in Mexico, USA & Colombia, corrected for inflammation excluding CRP > 5.0 ng/mL, SF < 15 µg/L ^3,4,5^ Low Ferritin %, High sTFR %, Vitamin A Deficiency % uncorrected for inflammation; ^3^ Low Ferritin defined as: SF <15 µg/L; ^4^ High sTFR defined as: sTFR > 8.3 mg/L; ^5^ Vitamin A Deficiency assessed using serum retinol < 0.70 µmol/L; ^6^ Elevated CRP > 5.0 ng/mL; ^7^ Anemia definition: Age < 11.99 years, Hb (g/dL) < 11.5; Age ≥ 12 years, Hb (g/dL) < 12.0 Anemia in Mexico using Hb adjusted for altitude and/or smoking, Anemia in the US using Hb adjusted for African American extraction, Anemia in Colombia using Hb adjusted for altitude and/or smoking and AA extraction; ^8^ Iron Deficiency Anemia definition %: % Iron deficiency anemia was defined as the presence of anemia using adjusted Hb along with low ferritin among those with CRP ≤ 5 mg/L; ^9^ Anemia associated with Iron Deficiency definition: % IDA ^8^ divided by % anemia ^7^. ^‡^ Ferritin (*n* = 733) and sTFR (*n* = 727) information for females aged 12 years and older. Vit A information (*n* = 3086) available in the US for both males and females aged 6 years and older.

**Table 2:** Characteristics associated with anemia in school aged children 5.00–14.99 years in Mexico and Colombia.

Characteristics	Mexico				Colombia			
	Anemia (%) *	UnAdj (95% CI)	** Adj OR (95% CI)	*p*-Value ^‡^	Anemia (%) *	UnAdj (95% CI)	** Adj OR (95% CI)	*p*-Value ^‡^
**Continuous**								
^1^ CRP (ng/mL)	-	-	1.0 (0.9–1.0)	0.16	-	-	1.0 (0.99–1.01)	0.23
**Categorical**								
^2^ Age (years)								
<12.0 years	12.0				4.4			
12.0–14.99 years	10.1	0.8 (0.5–1.3)	0.7 (0.5–1.2)	0.19	3.7	0.8 (0.6–1.1)	0.8 (0.5–1.0)	0.08
Sex								
Male	11.0				4.3			
Female	12.2	1.1 (0.8–1.6)	1.2 (0.9–1.7)	0.30	4.1	1.0 (0.8–1.3)	1.0 (0.7–1.3)	0.90
^3^ Race/Ethnicity								
Non Black					3.8			
Black	N/A	N/A	N/A	N/A	7.1	1.9 (0.4–2.7)	1.6 (1.2–2.3)	0.005
^4^ Asset								
All Other	11.1				3.3			
Poorest	13.0	1.2 (0.9–1.7)	1.1 (0.8–1.6)	0.55	6.7	2.1 (1.6–2.9)	1.8 (1.3–2.5)	0.0005
^5^ Low Ferritin %								
No	10.9				3.7			
Yes	14.8	1.4 (1.0–2.0)	1.5 (1.1–2.0)	0.02	9.3	2.7 (1.9–3.7)	2.7 (2.0–3.8)	<0.0001
^6^ Overweight								
No	12.6				4.3			
Yes	5.5	0.4 (0.2–0.8)	0.4 (0.2–0.8)	0.007	1.7	0.4 (0.2–1.0)	0.5 (0.2–1.2)	0.11
^7^ Stunting								
No	11.7				3.9			
Yes	11.6	1.0 (0.6–1.5)	0.9 (0.5–1.4)	0.51	6.3	1.7 (1.1–2.4)	1.6 (1.1–2.3)	0.02

* Percentage of SAC with anemia in each predictor group of interest ^‡^ *p* value is for association between the given factor and anemia in the multivariable model, null hypothesis for *p*-value is that Odds Ratio (OR) = 1 ** Adjusted for all other variables in the table, anemia OR is presented for a multivariable logistic model (outcome = anemia) that accounted for cluster study design: sample size available in each country for multivariable model—Mexico (*n* = 3644), Colombia (*n* = 8390). ^1^ CRP: C-Reactive Protein; used as a continuous variable; ^2^ Age categories based on 12 years as the average of menarche for girls in each of the two countries, grouped for analysis as Age < 12.0 and Age 12.0–14.99 years; ^3^ Grouped for analysis as Black and Non-Black; ^4^ Grouped for analysis as Poorest and All Other. Original categories per PCA Asset Index—Poorest, Poor, Average, Rich, Richest; ^5^ Low Ferritin defined as: Age ≥ 5 years, SF < 15 µg/L; ^6^ Grouped for analysis with Overweight = BAZ > 2 (Y/N); ^7^ Grouped for analyses with Stunting = HAZ < −2 (Y/N). N/A: No ethnicity information in MX.

**Table 3:** Characteristics associated with anemia among school-aged adolescent girls aged 12.00–14.99 years in the USA.

^†^ Characteristics	USA			
	Anemia (%) *	UnAdj (95% CI)	** Adj OR (95% CI)	*p*-Value ^‡^
**Continuous**				
^1^ CRP (ng/mL)	-	-	1.0 (0.99–1.04)	0.06
^2^ Age (years)	-	-	0.9 (0.4–1.8)	0.72
**Categorical**				
^3^ Race/Ethnicity				
Non Black	1.2			
Black	16.7	16.5 (5.5–49.5)	14.1 (4.7–42.1)	<0.0001
^4^ Asset				
All Other	3.0			
Poorest	7.9	2.8 (1.3–5.9)	1.3 (0.8–2.2)	0.33
^5^ Low Ferritin %				
No	2.2			
Yes	17.2	9.1 (4.5–18.4)	8.0 (3.0–21.3)	<0.0001
^6^ Overweight				
No	3.6			
Yes	3.9	1.1 (0.4–3.2)	0.8 (0.3–2.0)	0.63

* Percentage of school-aged adolescent girls with anemia in each predictor group of interest ^‡^ *p* value is for association between the given factor and anemia in the multivariable model, null hypothesis for *p*-value is that OR = 1 ** Adjusted for all other variables in the table, anemia OR is presented for a multivariable logistic model (outcome = anemia) that accounted for cluster study design: sample size available in the USA for multivariable model *n* = 697. ^1^ CRP: C-Reactive Protein; used as a continuous variable; ^2^ Age used as a continuous variable; ^3^ Grouped for analysis as Black and Non-Black; ^4^ Grouped for analysis as Poorest and All Other. Original categories per PCA Asset Index—Poorest, Poor, Average, Rich, Richest; ^5^ Low Ferritin defined as: Age ≥ 5 years, SF < 15 µg/L; ^6^ Grouped for analysis with Overweight = BAZ > 2 (Y/N) ^†^ There were no vitamin A deficient or stunted school-aged adolescent girls with anemia in the US.

## Supplementary Materials

The following are available online at http://www.mdpi.com/2072-6643/8/7/387/s1, Table S1. Summary of laboratory analytical methods for biomarkers of interest in Mexico, USA and Colombia, Table S2. Basic demographic characteristics of original & final (after applying exclusion criteria) datasets in Mexico and Colombia, Table S3. Anthropometric and biochemical characteristics of school aged children aged 5–14.99 years in the USA.

## Abbreviations

The following abbreviations are used in this manuscript:

SAC	School-Aged Children
ID	Iron Deficiency
OR	Odds Ratio
CI	Confidence Interval
YLD	Years Lived with Disability
IDA	Iron Deficiency Anemia
PSC	Pre-School Children
CDC	Centers for Disease Control and Prevention
WRA	women of reproductive age
Hb	Hemoglobin
CRP	C-reactive protein
AGP	α-1-acid glycoprotein
sTFR	soluble transferrin receptor
ENSANUT	Mexican National Health and Nutrition Survey 2006
NHANES	The National Health and Nutrition Examination Surveys
ENSIN	Encuesta Nacional de la Situaci ón Nutricional
WHO	World Health Organization
SES	socio-economic status
PCA	Principal component analysis
PIR	poverty income ratio
SE	standard error
IRB	institutional review board
