# Supplementary material for: Determinants of Anemia among School-Aged Children in Mexico, the United States and Colombia
Source: Nutrients. 2016 Jun 23;8(7):387. doi: 10.3390/nu8070387 (PMC4963863; doi:10.3390/nu8070387)
Supplement: Supplementary file 1 [file nutrients-08-00387-s001.docx]

Supplementary Materials: Determinants of Anemia among School-Aged Children in Mexico, the United States and Colombia

Sana Syed, O. Yaw Addo, Vanessa De la Cruz-Góngora, Fayrouz A. Sakr Ashour,
Thomas R. Ziegler and Parminder S. Suchdev

**Table S1.** Summary of laboratory analytical methods for biomarkers of interest in Mexico, USA
and Colombia.

| **Biomarkers** | **Countries** | | |
| --- | --- | --- | --- |
|  | **Mexico** | **USA** | **Colombia** |
| **Hemoglobin** | HemoCue  analyzer (HemoCue) | Beckman Coulter | HemoCue  analyzer (HemoCue) |
| **Ferritin** | Immunoassay method using commercial  Kits. (Behring Nephelometer 100 Analyzer) CV * 4.71% | Immuno-radiometry  In 2003: BioRad assay  Immuno-turbidimetry in 2004-6: Roche/Hitachi 912 clinical analyzer | Chemoluminescence (ADVIA Centaur,  Siemens) assay |
| **Soluble  transferrin receptor** | N/A | Immuno-turbidimetry (Roche/Hitachi 912 clinical analyzer) | N/A |
| **Retinol** | N/A | High Performance Liquid  Chromatography (Isocratic HPLC) | N/A |
| **CRP** | Nephelometry using ultrasensitive monoclonal antibodies (Behring Nephelometer 100 Analyzer) CV 4.2% | Latex-enhanced nephelometry (Behring Nephelometer) | Turbidimetric (ASC-180, Bayer Diagnostics) assay |

* Coefficient of variation (CV); N/A = Not Applicable.

**Table S2.** Basic demographic characteristics of original & final (after applying exclusion criteria) datasets in Mexico and Colombia.

| **Characteristics** | **Countries** | | | | | | | |
| --- | --- | --- | --- | --- | --- | --- | --- | --- |
|  | **Mexico ^¥^** | | | | **Colombia** | | | |
|  | **Original Dataset** | | **Final Dataset** | | **Original Dataset** | | **Final Dataset** | |
|  | ***n* *** | **% or  Mean (SE)** | ***n* *** | **% or  Mean (SE)** | ***n* *** | **% or  Mean (SE)** | ***n* *** | **% or  Mean (SE)** |
| Age (years) | 3708 | 9.1 (0.1) | 3660 | 9.1 (0.1) | 11239 | 10.5 (0.04) | 8573 | 9.9 (0.04) |
| Height (cm) | 3706 | 129.6 (0.5) | 3658 | 129.5 (0.5) | 11058 | 136.3 (0.2) | 8465 | 133.2 (0.2) |
| Weight (kg) | 3706 | 33.9 (1.1) | 3658 | 33.7 (1.1) | 11058 | 34.2 (0.2) | 8465 | 32.0 (0.2) |
| Sex % (Females) | 2147/3708 | 59.8% (1.6) | 2115/3660 | 59.5% (1.6) | 5,485/11,239 | 48.3% (0.6) | 4944/8573 | 57.4% (0.7) |

* Stated *n* of those SAC with age, height, weight and sex information available. Final datasets after study exclusion criteria applied per Figure 1 (excluded from final analysis if pregnant or if data not available for hemoglobin, CRP or ferritin); ^¥^ In Mexico, per country survey design, heights and weights were available on a representative subset of randomly selected children.

**Table S3.** Anthropometric and biochemical characteristics of school aged children aged
5–14.99 years in the USA.

| **Characteristics** | **USA/*n* = 3543** | |
| --- | --- | --- |
|  | ***n* *** | **% (SE of %) or Mean (SE)** |
| *Demographics* |  |  |
| Age in years | 3543 | 10.3 (0.1) |
| Age |  |  |
| <12.0 years | 2063/3543 | 66.7% (1.0) |
| 12.0–14.99 years | 1480/3543 | 33.3% (1.0) |
| Sex (Females) % | 1790/3543 | 48.2% (1.1) |
| Race/Ethnicity |  |  |
| Black | 1173/3543 | 15.2% (1.8) |
| Non-Black | 2370/3543 | 84.8% (1.8) |
| Asset |  |  |
| Poorest | 831/3419 | 16.0% (1.3) |
| All Other | 2588/3419 | 84.0% (1.3) |
| *Nutrition/Growth* |  |  |
| Stunting % (HAZ < −2) | 36/3507 | 0.9% (0.2) |
| Wasting % (BAZ < −2) | 42/3507 | 1.2% (0.3) |
| Overweight % (BAZ > 2) | 740/3507 | 18.9% (1.4) |
| Obese % (BAZ > 3) | 224/3507 | 4.7% (0.6) |
| *Biochemical markers* |  |  |
| Hemoglobin (g/dL) | 3543 | 13.6 (0.5) |
| Hemoglobin (g/dL) adjusted for altitude &/or ^2^ smoking | N/A | N/A |
| C-Reactive Protein (ng/mL) | 3543 | 1.7 (0.1) |
| ^1^ Iron Deficiency % | ^‡^ 104/971 | 8.8% (1.0) |
| ^2^ Low Ferritin% | ^‡^ 110/1056 | 8.4% (1.0) |
| ^3^ High sTFR% | ^‡^ 90/1038 | 7.8% (1.2) |
| ^4^ Vitamin A Deficiency % | ^‡^ 25/3086 | 0.6% (0.1) |
| ^5^ Elevated C-Reactive Protein (ng/mL) % | 268/3543 | 6.6% (0.6) |
| ^6^ Anemia % | 106/3543 | 1.5% (0.2) |
| ^7^ Iron Deficiency Anemia using adjusted Hb% | ^‡^ 18/971 | 1.3% (0.4) |

* Reported *n* is of actual sampled population. Reported % are weighted per the survey design, Abbreviations: Height for Age *z* score—HAZ; Body Mass Index for Age *z* score—BAZ; soluble transferrin receptor—sTfR. ^1^ Iron Deficiency % assessed using serum ferritin (SF), corrected for inflammation excluding CRP > 5.0 ng/mL, SF < 15 µg/L, ^2,3,4^ Low Ferritin %, High sTFR %, Vitamin A Deficiency % uncorrected for inflammation; ^2^ Low Ferritin defined as: SF <15 µg/L; ^3^ High sTFR defined as: sTFR > 8.3 mg/L; ^4^ Vitamin A Deficiency assessed using serum retinol < 0.70 µmol/L;
^5^ Elevated CRP > 5.0 ng/mL; ^6^ Anemia definition: Age < 11.99 years, Hb (g/dL) < 11.5; Age ≥ 12 year,
Hb (g/dL) < 12.0. Anemia in Mexico using Hb adjusted for altitude &/or smoking, Anemia in the US using Hb adjusted for African American extraction, Anemia in Colombia using Hb adjusted for altitude &/or smoking and AA extraction; ^7^ Iron Deficiency Anemia definition %: % Iron deficiency anemia was defined as the presence of anemia using adjusted Hb along with low ferritin among those with CRP ≤ 5 mg/L; ^‡^ Ferritin (*n* = 1056) and sTFR (*n* = 1038) information available in the US for children aged 5 years both sexes and females aged 12 years and older. Vit A information (*n* = 3086) available in the US for both males and females aged 6 years and older. N/A = Not Applicable.
